# Monitoring program design for data‐limited marine biogenic habitats: A structured approach

**DOI:** 10.1002/ece3.5261

**Published:** 2019-05-20

**Authors:** Tse‐Lynn Loh, Stephanie K. Archer, Anya Dunham

**Affiliations:** ^1^ Quest University Canada Squamish British Columbia Canada; ^2^ Fisheries and Oceans Canada, Pacific Biological Station Nanaimo British Columbia Canada

**Keywords:** benthic assessment, benthic indicators, biogenic habitat, data‐limited habitats, foundation species, monitoring protocol, survey methodology

## Abstract

Marine biogenic habitats—habitats created by living organisms—provide essential ecosystem functions and services, such as physical structuring, nutrient cycling, biodiversity support, and increases in primary, secondary, and tertiary production. With the growing trend toward ecosystem approaches to marine conservation and fisheries management, there is greater emphasis on rigorously designed habitat monitoring programs. However, such programs are challenging to design for data‐limited habitats for which underlying ecosystem processes are poorly understood. To provide guidance in this area, we reviewed approaches to benthic assessments across well‐studied marine biogenic habitats and identified common themes related to indicator selection, sampling methods, and survey design. Biogenic habitat monitoring efforts largely focus on the characteristics, distribution, and ecological function of foundation species, but may target other habitat‐forming organisms, especially when community shifts are observed or expected, as well as proxies of habitat status, such as indicator species. Broad‐scale methods cover large spatial areas and are typically used to examine the spatial configuration of habitats, whereas fine‐scale methods tend to be laborious and thus restricted to small survey areas, but provide high‐resolution data. Recent, emerging methods enhance the capabilities of surveying large areas at high spatial resolution and improve data processing efficiency, bridging the gap between broad‐ and fine‐scale methods. Although sampling design selection may be limited by habitat characteristics and available resources, it is critically important to ensure appropriate matching of ecological, observational, and analytical scales. Drawing on these common themes, we propose a structured, iterative approach to designing monitoring programs for marine biogenic habitats that allows for rigorous data collection to inform management strategies, even when data and resource limitations are present. A practical application of this approach is illustrated using glass sponge reefs—a recently discovered and data‐limited habitat type—as a case study.

## 1 INTRODUCTION

Marine biogenic habitats, such as coral reefs, sea grass beds, and kelp forests, are created by living organisms (foundation species) that form emergent three‐dimensional structures creating niche space for other species (Bruno & Bertness, 2001; Dayton, 1972; Roberts, Johnston, & Poore, 2008) and providing important ecological functions. For example, reef‐building corals and sponges contribute to biogeochemical cycling (Dunham, Archer, et al., 2018; Kahn, Yahel, Chu, Tunnicliffe, & Leys, 2015; Wild et al., 2004), while photosynthesizing species support food webs and provide “blue carbon” storage (Cebrián, Pedersen, Kroeger, & Valiela, 2000; Duarte & Krause‐Jensen, 2017; Miller, Reed, & Brzezinski, 2011). Further, in areas dominated by soft, unstable substrates, biogenic structures create stable settlement habitats and thus facilitate larval recruitment and survival (Lindsey, Altieri, & Witman, 2006). Seagrass and mangrove habitats are essential nursery grounds for juvenile fish and invertebrates (Mumby et al., 2004; Unsworth, Nordlund, & Cullen‐Unsworth, 2018). In addition to the wide range of ecosystem functions biogenic habitats contribute to, they provide a wide range of services. Globally, coral reef fisheries contribute critical animal protein and estimated annual benefits of US$5.7 billion (Cesar, Burke, & Pet‐soede, 2003; Whittingham, Campbell, & Townsley, 2003). Biogenic structures also defend against erosion and waves, reducing human death toll and infrastructure damage from natural disasters (Danielsen et al., 2005; Das & Vincent, 2009). Understanding the ecology and functioning of biogenic habitats has shed light on their vital importance for the continued conservation of natural resources and associated ecosystem services.

As resource management moves toward holistic, habitat‐based approaches such as ecosystem‐based fisheries management (Pikitch et al., 2004), there is a growing need for management actions that promote healthy biogenic habitats. These management actions require ecological monitoring with clear research questions, appropriate indicators, and a well‐designed data collection process to produce robust data and useful outcomes (Underwood & Chapman, 2013). A recently developed framework for biological monitoring (Reynolds, Knutson, Newman, Silverman, & Thompson, 2016) offered an overarching view of the steps required for successful monitoring programs and emphasized the importance of linkages among various planning decisions. However, for data‐limited biogenic habitats, designing monitoring programs can be challenging. The structure and functioning of such habitats and underlying ecosystem‐level processes (e.g., spatial extent, magnitude of natural variability in abundance and distribution of foundation species, species–habitat associations) are not well understood, often due to these habitats being remote and/or deep, limiting accessibility, and increasing monitoring costs. These limitations may jeopardize effective management and conservation of these habitats, many of which are threatened by human activities (Rossi, Bramanti, Gori, & Orejas Saco del Valle, 2017). A clear road map for designing robust, efficient monitoring programs in the face of data and resource limitations is required.

We reviewed recent publications (2012–17; Appendix S1) to obtain an overview of benthic assessment and monitoring approaches across a range of relatively well‐studied marine biogenic habitats. Next, we identified common themes relevant for all habitat types reviewed and, drawing upon these themes, developed a systematic approach for establishing monitoring programs for data‐limited biogenic habitats. Finally, we illustrated a practical application of this approach using glass sponge reefs—a recently discovered and data‐limited habitat type—as a case study (Box ).

Box 1 **Case study: Glass sponge reefs off British Columbia, Canada**

**Figure 1:**
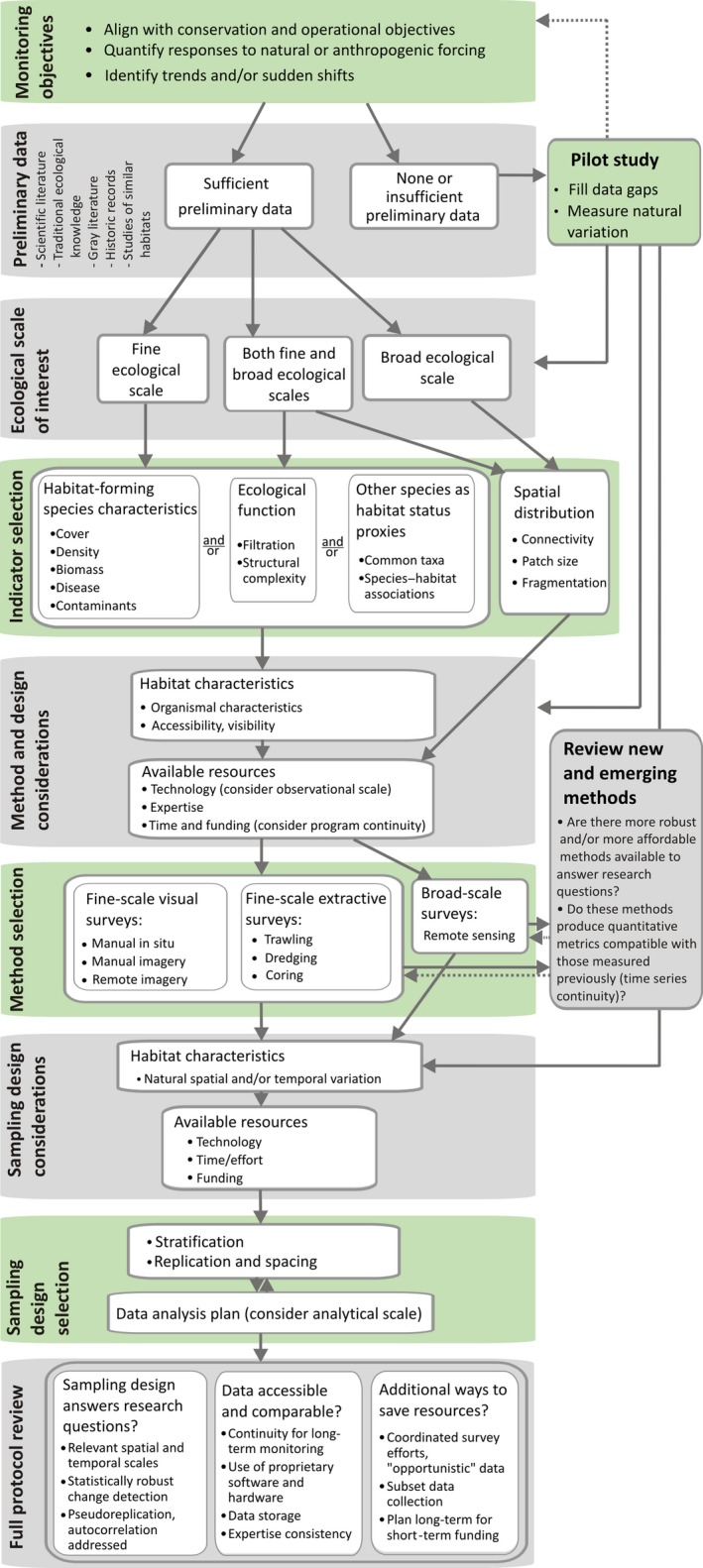
Flowchart illustrating the development of a monitoring protocol for marine biogenic habitats. Gray boxes contain considerations (to facilitate evaluation of available data, methods, and protocols), and green boxes denote decisions. Key times for iteration back through earlier steps are denoted by the return dashed arrows

1 Extant glass sponge reefs were first discovered in the late 1980s (Conway, Barrie, Austin, & Luternauer, 1991) and are now known to extend from southern British Columbia, Canada to southwestern Alaska, United States. These reefs are recognized as a globally rare ecosystem and are already impacted by human activities such as trawling (Conway, Krautter, Barrie, & Neuweiler, 2001). Thus, scientific advice for protection and monitoring was urgently required despite the incomplete ecological knowledge of the system. In 2015, Fisheries and Oceans Canada (DFO) designated nine glass sponge reef complexes in the Strait of Georgia as protected areas where all bottom‐contact fishing activities are prohibited. Below, we illustrate how science advice (described in Dunham, Archer, et al., 2018, Dunham, Mossman, et al., 2018) for the protection initiative was developed despite data limitations following the monitoring protocol flowchart (see Figure 1).

*Objective*: To establish a baseline for the status of structural habitat, biodiversity, and ecosystem function in the nine glass sponge reefs and recommend a monitoring approach capable of detecting temporal trends in these attributes.

*Preliminary data* were gathered, and pilot studies were conducted in 2012–2013 to develop quantitative methods for assessing reef status. Pilot surveys also provided the ecological baseline for reef status prior to spatial protection. Previously, the spatial extent of the glass sponge reefs was delineated using acoustic remote sensing techniques (e.g., Conway, Barrie, & Krautter, 2005) and reef condition was assessed qualitatively (Cook, Conway, & Burd, 2008). Observational studies indicated that the reef‐building sponges, *Aphrocallistes vastus* and *Heterochone calyx* (Porifera, Hexactinellida), are slow‐growing and unlikely to quickly recover from large disturbances (Dunham et al., 2015; Kahn, Vehring, Brown, & Leys, 2016; Leys & Lauzon, 1998).

*Ecological scale of interest*: Because the broad‐scale spatial distribution of glass sponge reefs in the Strait of Georgia is unlikely to change within the management timescale, we considered ecological patterns and processes of interest at the scale of individual reefs, mostly at fine spatial scales. The expansion of reef boundaries from sponge growth may necessitate the use of broad‐scale methods and associated indicators (e.g., seascape patch metrics) to track spatial changes in the future.

**Figure 2:**
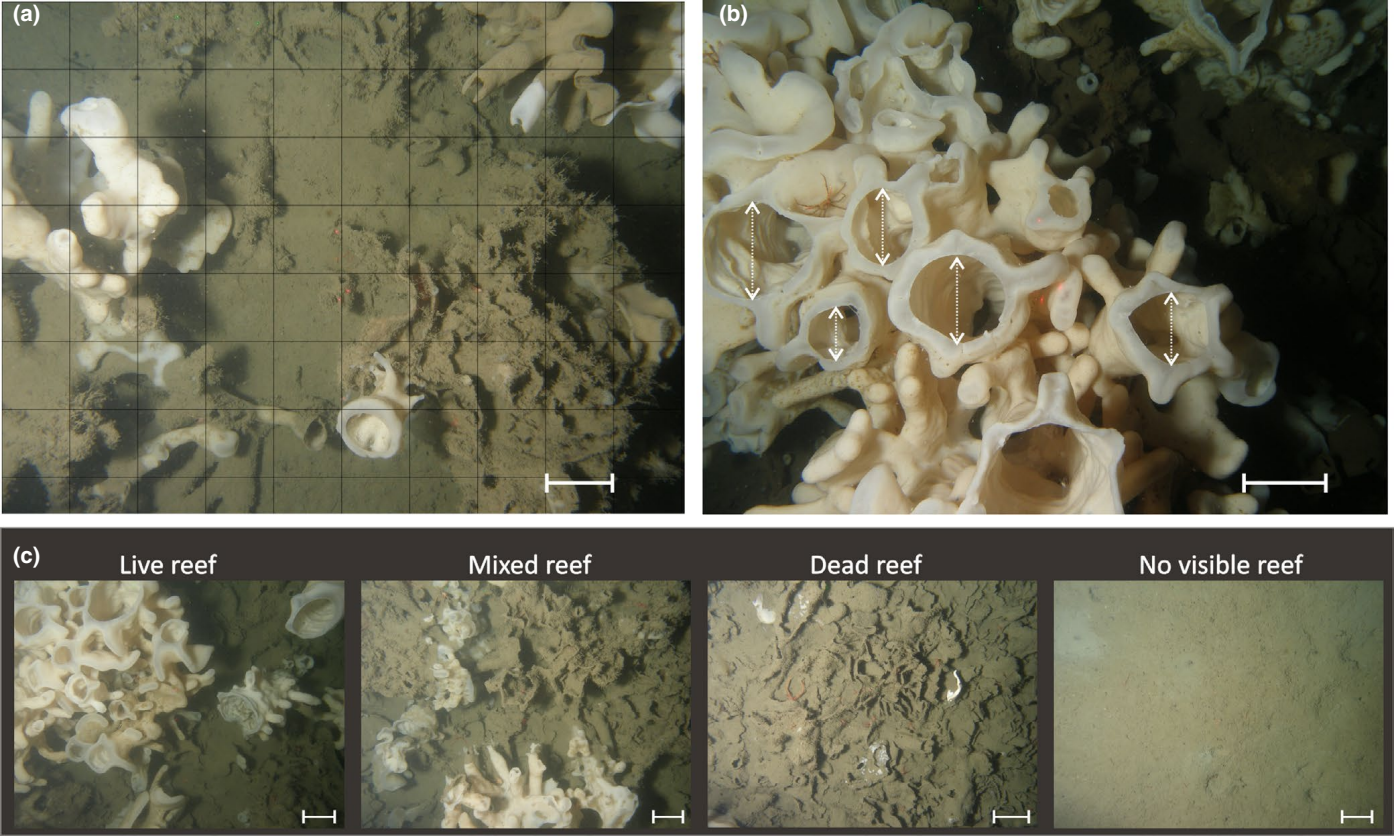
Examples of remote imagery used to derive quantitative metrics of glass sponge reef status: (a) Percent live reef‐building sponge cover was calculated from still images by overlaying a 10 × 10 cm grid; (b) oscula size and density were measured using still images (all oscula were counted, but only camera‐facing ones were measured); and (c) relative proportions of four habitat categories were calculated from video (collected along line transects) by assigning one habitat category to each 10‐s video segment. Scale bars are 10 cm

*Indicator and metric selection*: The reef‐building sponges often grow so densely that distinguishing individuals is impractical or impossible (Dunham, Archer, et al., 2018). Additionally, the relationship between sponge benthic cover and biomass is not known, and sampling such slow‐growing and slow‐to‐recover sponges to elucidate this relationship could damage ecosystem health. Therefore, instead of density or biomass, we measured sponge abundance using (a) percent cover calculated from still images and (b) the relative proportions of four habitat categories (no visible reef, dead reef, mixed reef, and live reef) from video (Figure 2a,c; methods described in detail in Dunham, Archer, et al., 2018).

As suspension feeders, sponges filter water to capture particulate food and expel filtered water through oscula (Kahn et al., 2015). Oscula density and area—both measured from still images (Figure 2b)— were proposed as metrics of ecosystem function representing sponge filtration rate.

**Figure 3:**
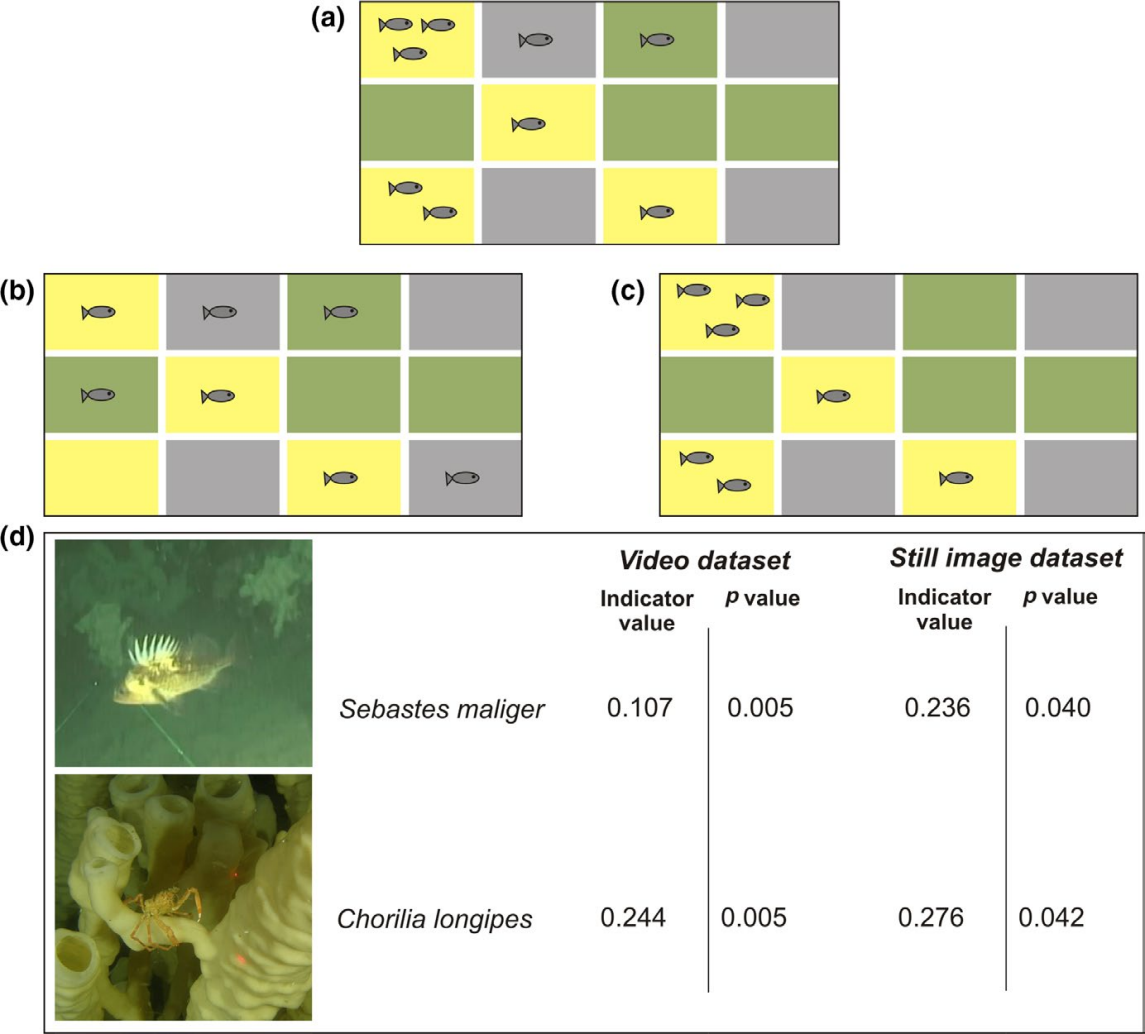
Using proxies for biogenic habitat attributes: an example of species‐habitat associations in the glass sponge reef case study. Indicator species analysis (Dufrêne & Legendre, 1997) combines measures of fidelity (the probability of finding a species in sites of a given a habitat type) and specificity (the probability a site belongs to a habitat type given that the species is there) into a single index with a maximum value of 1. For example, the taxon in panel (a) has an indicator value of 0.78 for the yellow habitat, and 0.03 for both the gray and green habitats. The taxon in panel (b) is not a good indicator while the taxon in panel (c) is a perfect indicator. On the sponge reefs, several taxa showed significant associations with live reef in both video and still image analysis (d); these taxa were recommended as indicators. Strong associations between rockfish and live reef habitat led to subsequent research evaluating the frequency of soniferous fish calls as measured by passive acoustics as a proxy for reef habitat status (Archer et al., 2018)

Indicator species analysis (De Cáceres & Legendre, 2009; Dufrêne & Legendre, 1997) was completed for community data gathered through both video and still image annotation (see Dunham, Mossman, et al., 2018 for detailed methods). Several taxa had significant associations with specific habitat types in both video and still images and were thus suggested as indicators for that habitat type: *Sebastes maliger* and *Chorilia longipes* for live reef, *Munida quadrispina* and *Pandalus platyceros* for visible (i.e., live or dead intact) reef, and Ophiuroidea for no visible reef (Figure 3).

*Method considerations*: While acoustic remote sensing methods can map the geologic sponge reef structure, they cannot distinguish between live, dead, and dead‐and‐buried reefs or reef areas (Chu & Leys, 2010). The majority of sponge reefs occur in waters with relatively high visibility below safe SCUBA diving limits. Therefore, remote visual sampling with an ROV was used for pilot studies and was recommended for future monitoring.

*Reviewing new and emerging methods*: Strong association between rockfish and live reef habitat led to the hypothesis that passive acoustics may offer an efficient monitoring tool, as rockfish are generally known to be soniferous (Širović, Cutter, Butler, & Demer, 2009). The frequency of fish calls can be an indicator of the status of the fish community on sponge reefs, although more research is needed to determine whether it can be used as a proxy for habitat status (Archer et al., 2018).

*Sampling design considerations and selection*: As the spatial heterogeneity of live glass sponge cover can be high (Chu & Leys, 2010), for the pilot study, we employed a stratified random design where each reef was divided into roughly equal sized sections (*n* = 2–11 per reef) and one 500 m line transect was randomly placed within each section. Most of the survey effort occurred within known reef polygons delineated using remote sensing, with a subset of transects extending beyond reef edges to ground truth and refine present reef boundaries. A combination of fixed and random transects was recommended for future monitoring. Fixed transects will allow trends in relative sponge abundance to be assessed, while random transects are more likely to capture impacts from localized stressors (e.g., fishing due to noncompliance). Given the low rates of sponge growth and recovery, a monitoring survey frequency of 5–10 years was recommended.

*Uncertainties*,* limitations*,* and future research needs*: Current remote visual survey methods limit our estimation of habitat complexity, a key ecological function. Because monitoring has just started, it may take a number of years to quantify natural variation sufficiently to distinguish ecosystem responses to environmental impacts from seasonal variation. Incorporating repeat transects into future survey designs will allow for the estimation of measurement error. Seascape patch metrics have not yet been developed for the sponge reefs, but recent progress made in delineating reef patches by combining visual survey and remote acoustic data (DFO, 2018) may assist in developing seascape metrics in the future. Finally, targeted research on sponge larval ecology and recruitment, as well as the resilience and recovery of individual sponges and reefs, will assist in refining status indicators and developing composite indices to aid future monitoring.

## 2 BIOGENIC HABITAT MONITORING PROTOCOL

Across biogenic habitat types, assessment and monitoring efforts share the following common themes: defining study objectives, assembling preliminary data, determining scale of interest, selecting indicators, determining study methods and sampling design, and full protocol review. In the sections that follow, we summarize key considerations and decisions under each theme and arrange them into a flowchart (Figure 1)—a systematic approach we recommend for establishing monitoring programs for data‐limited biogenic habitats.

### 2.1 Objectives

Appropriate objective setting is crucial for effective ecological monitoring (Reynolds et al., 2016 and studies cited therein). In biogenic habitats, common *conservation objectives* (or “fundamental objectives” sensu Reynolds et al., 2016) are to conserve the habitat, its ecosystem function, and associated biodiversity. From there, *operational objectives* (or “means” objectives sensu Reynolds et al., 2016) must define a measurable desired state, threshold value, amount of change, or trend for a particular habitat characteristic (*ecological indicator*) to be monitored. In general, biogenic habitat monitoring programs assess habitat status over space and/or time (Perkins, Foster, Hill, Marzloff, & Barrett, 2017). Although specific monitoring objectives vary, the overall goal is typically to assess changes in ecological indicators by using appropriate quantitative biological, chemical, or physical measurements (*metrics*) and to identify the drivers of change (shifts or trends) observed. Potential drivers of change fall into one or more broad categories: natural or anthropogenic pressures (Bo et al., 2014; Kawamura et al., 2014), broad‐scale stressors such as climate change (Sahade et al., 2015), and specific management measures (Bégin et al., 2016; Howarth et al., 2015).

### 2.2 Preliminary data

After objectives are identified, all available information about the habitat is gathered, including qualitative descriptions, traditional ecological knowledge, historic records of the area, and data from studies in similar ecosystems. A conceptual model of the ecosystem may be sketched to clarify what is known (and not known) about the ecosystem (Reynolds et al., 2016). Preliminary data provide the first characterization of the habitat, identifying the foundation species and major ecological relationships (Moura et al., 2016), and help with determining ecological scale of patterns or processes of interest (fine, broad, or both). Preliminary data also inform method and survey design selection and help determine level of replication for sufficient statistical power to detect change at ecologically relevant effect sizes (Kipson et al., 2011; Underwood & Chapman, 2013). Assembling preliminary data also serves to highlight knowledge gaps; when information is unavailable or insufficient to guide survey design, as is the case in many data‐limited systems, a pilot study is recommended as well‐designed surveys eventually save time, money, and effort.

### 2.3 Indicator selection

Monitoring indicators must be aligned with conservation and operational objectives, be ecologically relevant, sensitive to potential stressors or management actions (Gill et al., 2011), and, because of logistical restrictions, easy to measure consistently. Our literature review (Appendix S1) revealed four clusters of indicators and associated metrics under the following broad habitat attributes: (a) habitat‐forming species’ characteristics, (b) habitat‐forming species’ spatial distribution, (c) habitat‐forming species’ ecological function, and (d) other species as proxies for habitat status (Table 1).

**Table 1:** Summary of attributes, representative indicators, and associated metrics used for biogenic habitat assessments, with corresponding survey methods. Full list of studies reviewed can be found in Appendix S1

Habitat attribute	Indicator examples	Metric examples	Survey methods							
			Broad scale					Fine scale		
			Satellite imaging	Aerial photography	Airborne LiDAR	Airborne hyperspectral imaging	Remote acoustics	Manual in situ census and measurements	Manual or remote (ROV/AUV) imagery	Extractive (trawling, dredging, coring)
Habitat‐forming species characteristics	Foundation species and/or other habitat‐forming species abundance	Live cover			x (coral only, fluorescence LiDAR)	x		x	x	
		Density						x	x	x
		Biomass								x
	Foundation species and/or other habitat‐forming species condition	Disease incidence						x	x	x
		Coral bleaching		x				x	x	
		Contaminant concentration								x
	Habitat‐forming species community structure	Select species presence				x (for macroalgal groups)		x	x	x
Spatial distribution	Habitat configuration	Patch area, proximity, connectivity	x	x	x	x	x			
	Patch shape complexity	Fractal dimension			x					
Ecological function	Filtration rate	Filtration unit density						x	x	
	Habitat provision	Rugosity, structural complexity			x			x	x (from photogrammetry)	
	Biodiversity support	Species richness/diversity						x	x	x
Other species as habitat status proxies	Common taxa	Select species abundance						x	x	x
	Indicator species									

#### 2.3.1 Habitat‐forming species characteristics

Data on the identity and abundance of benthic organisms, especially of the foundation species, are commonly collected in biogenic habitat monitoring programs (Facon et al., 2016; Fatoyinbo & Simard, 2013; Gorman, Bajjouk, Populus, Vasquez, & Ehrhold, 2013; Short et al., 2014). The metrics used to assess foundation species abundance include live cover, density, and biomass. Live benthic cover is recorded in situ or calculated from video or still images as the proportion of the benthic area assessed that is occupied by the taxon of interest, and usually does not require sample collection (Jokiel et al., 2015). However, live benthic cover metric does not account for three‐dimensional structural complexity, and density (individuals per unit area) does not account for the differences in sizes between individuals. Biomass (mass per unit area) provides the most information of the three metrics, but requires the collection of biological material (Gorman et al., 2013), at least until reliable mathematical relationships between area covered or organism size and biomass can be established (McMurray, Blum, & Pawlik, 2008). Sample collection is unlikely to be carried out for severely impacted or slow‐growing foundation species, limiting the number of systems where biomass data can be collected.

While it is possible to only survey the abundance of foundation species (Fatoyinbo & Simard, 2013; Gorman et al., 2013), considering other habitat‐forming organisms provides a more complete picture. Many stable communities are maintained not by a single habitat‐forming species, but by multiple, co‐occurring foundation species (Angelini, Altieri, Silliman, & Bertness, 2011). For example, although live coral cover is commonly used to represent coral reef status, coral cover is very low across the Caribbean (Schutte, Selig, & Bruno, 2011). While corals constructed the original reef structure, sponges are presently the dominant habitat‐forming organisms (Loh, McMurray, Henkel, Vicente, & Pawlik, 2015). Therefore, disregarding sponge abundance would incompletely represent the Caribbean reef habitat. One solution is to take a community‐based approach to monitoring; for example, temporal comparisons of habitat‐forming species community structure are used to describe trends, estimate natural variation, and track recovery from previous detrimental impacts (de Bakker et al., 2017; Perkins et al., 2017; Short et al., 2014).

Indicators of habitat‐forming species condition are often assessed using stress response metrics, such as incidences of physical damage from bottom trawling (Fosså, Mortensen, & Furevik, 2002), disease incidence (Angermeier, Glöckner, Pawlik, Lindquist, & Hentschel, 2012; van Woesik & Randall, 2017), stress responses such as coral bleaching (Oliver, Berkelmans, & Eakin, 2018), and contaminant concentrations in organism tissues (Roberts et al., 2008).

#### 2.3.2 Spatial distribution

On a broad spatial scale, spatial distribution of habitats and subhabitats can also be used as indicators, as with global mangrove and seagrass (Giri et al., 2011; Waycott et al., 2009). Over the past three decades, the field of seascape ecology has emerged, whereby landscape ecology approaches are applied to quantify structure and delineate patch‐based models in marine habitats. Seascape patch metrics applied in several intertidal and subtidal habitats to date include patch size and shape, connectivity, and fragmentation (Boström, Pittman, Simenstad, & Kneib, 2011; Wedding, Lepczyk, Pittman, Friedlander, & Jorgensen, 2011).

#### 2.3.3 Ecological function

The dominant ecological functions linked to foundation species—for example, nutrient cycling, habitat provision, and primary, secondary, and tertiary production—can also be used as indicators. Metrics include filtration rates in deep‐water sponge assemblages (Dunham, Archer, et al., 2018; Kahn et al., 2015), rates of primary production of mangroves and kelp (Bouillon et al., 2008; Miller et al., 2011; Reed, Rassweiler, & Arkema, 2008), and structural complexity of coral reefs (González‐Rivero et al., 2017; Storlazzi, Dartnell, Hatcher, & Gibbs, 2016). Ecological function indicators may also address sublethal stress responses, such as reduced coral recruitment levels and sea grass photosynthesis in waters with elevated suspended sediment concentrations (Dikou & van Woesik, 2006; Goodman, Moore, & Dennison, 1995) and reduced mussel feeding rates during algal blooms (Tracey, 1988).

#### 2.3.4 Other species as proxies of biogenic habitat status

Ecological surrogates such as indicator, umbrella, and proxy species are widely used in ecological monitoring (Caro, 2010; Siddig, Ellison, Ochs, Villar‐Leeman, & Lau, 2016). For example, habitat features are used as surrogates for species abundance, provided their relationships have been quantified and are strong and predictive (Ferrari et al., 2018 and references therein). In assessing and monitoring biogenic habitat status, the inverse approach can be applied: The abundances of taxa known to have a strong association with biogenic habitat attributes that are more difficult or costly to measure, and that are sensitive to potential ecosystem impacts, can be quantified (Zacharias & Roff, 2001). For example, the presence or absence of certain polychaete species or shifts in polychaete community structure can indicate the presence of chemical pollutants across a wide range of marine systems (Dean, 2008). If associations and functional relationships between species are not well elucidated, monitoring efforts are sometimes focused on most abundant or widely distributed taxa (Perkins et al., 2017). This strategy avoids zero‐inflated data for comparisons, a common issue in studies of community ecology (Martin et al., 2005), but makes the assumption that the abundance of a taxon is proportional to its effect on ecosystem function and correlated with ecosystem status.

### 2.4 Survey method considerations and selection

Broad‐scale methods typically rely on remote sensing techniques and are used to examine the spatial configuration of the habitat, whereas fine‐scale methods examine habitat characteristics (see Table 1). Each method has its limitations, advantages, and disadvantages (Table 2).

**Table 2:** Applicability, advantages, and disadvantages of broad‐ and fine‐scale survey methods for biogenic habitats

Method	Habitat requirements	Habitat examples	Advantages	Disadvantages
Broad scale				
Satellite imaging	Shallow, clear water	Seagrass, mangrove, coral reef	Cover very large areas, global coverage possible Images free or cheap to obtain	Require cloudless conditions Depth‐limited Require specialized software and data processing skills
Aerial photography	Shallow, clear water	Mussel bed, coral reef	Cover large areas	Coarse data resolution Require cloudless conditions Only for habitats clearly visible at the sea surface Data collection expensive
Airborne LiDAR	Shallow, clear water	Mangrove, coral reef, macroalgae	Cover large areas Able to measure structural complexity	Depth‐limited Data collection expensive Equipment may be expensive Require specialized software and data processing skills Mostly unable to measure benthic cover
Airborne hyperspectral imaging	Shallow, clear water	Coral reef, macroalgae	Cover large areas Finer data resolution than satellite imaging	Depth‐limited Data collection expensive Equipment may be expensive Require specialized software and data processing skills Still at proof‐of‐concept stage
Remote Acoustics	Differences in sound reflectance among benthic categories	Seagrass, oyster reef, coral reef, deep coral, soft sediment	Cover large areas Can be used in turbid waters Can survey deep habitats	Equipment may be expensive Proprietary hardware and software Coarse data resolution
Fine scale				
Manual in situ census and measurements	Shallow, adequate visibility	Seagrass, oyster reef, mussel bed, mangrove, coral reef, sponge, macroalgae, rocky reef	Cheap equipment Data collection relatively inexpensive High data resolution	Require data collectors with taxonomic expertise Fieldwork‐intensive Data need to be manually transcribed from datasheets
Manual imagery	Shallow, good visibility	Seagrass, coral reef, temperate reef, sponge, macroalgae, rocky reef	Permanent data records Photomosaics option Less time in the field compared to in situ data collection	Require large data storage capacity Manual annotation time‐intensive Image resolution may be insufficient for accurate identification Equipment more expensive than for manual in situ surveys
Remote (ROV/AUV) imagery	Good visibility	Seagrass, coral reef, deep‐water coral, temperate reef, deep sponge, macroalgae	Can be used in deep habitats Cover larger area than manual methods Permanent data records Photomosaics option Less underwater time compared to manual methods	Require large data storage capacity Manual annotation time‐intensive Image resolution may be insufficient for accurate identification Equipment may be expensive Data collection expensive
Extractive (trawling, dredging, coring)	Able to recover from destructive sampling in a timely manner	Deep sponge, oyster reef, tubeworm reef, deep coral	Can be used in deep habitats Not dependent on visibility Can identify organisms to species Permanent specimen record “Free” data from fisheries bycatch	Destructive sampling may not be appropriate for many biogenic habitat types Equipment may be expensive Data collection expensive

The choice of sampling method is often driven by the characteristics and growth form of the foundation species. Data on slow‐growing or protected species such as reef‐building corals are generally collected through in situ manual recording or with imagery (Jokiel et al., 2015). In shallow (within SCUBA diving limits) habitats with safe working conditions, manual surveys are frequently employed. Yet, using photographs and/or video to survey benthic habitats may enable covering larger spatial areas compared to manual methods. Another major advantage of using imagery is the creation of a permanent record for data verification and for further data extractions (McMurray, Henkel, & Pawlik, 2010). In deep‐water biogenic habitats, remotely operated (ROVs) or autonomous underwater vehicles (AUVs) mounted with cameras are deployed for data collection (Armstrong et al., 2006; Bo et al., 2014). For both manual and remotely operated visual methods, sufficient visibility is required. Benthic cover is measured using the line intercept method, points along the line transect or within quadrats, by tracing benthos outlines in photographs or by visually estimating abundance according to a ranked scale (Jokiel et al., 2015).

Fast‐growing and abundant benthic species such as seagrasses, macroalgae, and bivalves can be destructively sampled by extractive (manual or excavating) methods for further examination, including taxonomic identification and determining biomass and biomarker levels (Molina Hernández & van Tussenbroek, 2014; Schulte, Lipcius, & Burke, 2018; Tsiamis et al., 2013). Extractive methods can also be used to sample slow‐growing species where visual census methods are not feasible, such as in turbid waters (Moura et al., 2016). However, in all cases, a thorough assessment of the benefit of sampling against the destruction of foundation species must be made and cumulative impacts of multiple sampling events must be taken into consideration. It can be difficult to accurately and consistently estimate the area sampled using extractive methods to calculate density or benthic cover. Abundance indices such as catch per unit effort are used instead, or indicators are restricted to species distribution and richness (Durán Muñoz et al., 2011; Murillo et al., 2012).

### 2.5 New and emerging methods in benthic assessments

Advances in survey technology and methodological improvements are blurring the distinction between broad‐ and fine‐scale assessments, moving toward capturing data over sizeable spatial areas at a high resolution and increasing data processing capacity to expand the amount and types of data collected (Hamylton, 2017). Fluorescence imaging LiDAR can detect fluorescent proteins in live corals, distinguishing them from dead corals, and thus be used to map coral cover when combined with satellite images (Sasano, Imasato, Yamano, & Oguma, 2016). Airborne hyperspectral sensors have potential for mapping live benthic cover in relatively shallow habitats with sufficient water clarity (Casal, Kutser, Domínguez‐Gómez, Sánchez‐Carnero, & Freire, 2013; Joyce, Phinn, & Roelfsema, 2013). Beyond cover, structure‐from‐motion (SfM) photogrammetry addresses structural complexity through the construction of three‐dimensional models of benthic habitat from several overlapping two‐dimensional images, often using stereo‐cameras (Burns, Delparte, Gates, & Takabayashi, 2015; Ferrari et al., 2016; Leon, Roelfsema, Saunders, & Phinn, 2015; Raoult et al., 2016).

In tandem with these emerging technologies, data storage capabilities and computing power have advanced immensely. Compact camera memory cards can support photographing entire study sites to construct high‐resolution photomosaics (Edwards et al., 2017; Pizarro, Friedman, Bryson, Williams, & Madin, 2017), in contrast to distributing survey quadrats across a study site in an attempt to represent the site accurately. Advances in machine learning and computing power have resulted in the improved accuracy of semi‐ and fully automated annotation to score benthic cover using points or perimeter tracing; these approaches offer substantial time savings over manual annotation (Beijbom et al., 2015; González‐Rivero et al., 2014; Teixidó et al., 2011).

### 2.6 Sampling design considerations and selection

Sampling design considerations can be broadly divided into those driven by habitat characteristics and by the resources available.

#### 2.6.1 Habitat‐driven design aspects

Benthic organisms are often nonuniform in their distribution and abundance and thus form a system with naturally high spatial variation (Underwood & Chapman, 2013). As such, surveys must implement sufficient spacing between samples to reduce pseudoreplication and autocorrelation (Gill et al., 2011; Underwood & Chapman, 2013). This is often challenging in data‐limited marine systems due to the insufficient understanding of the distribution and abundance of habitat types at both broad and fine scales, especially when a significant change in habitat distribution occurred between sampling times. Consequently, it can be challenging to distinguish changes in habitat status from natural variation and appropriately match the survey scale to the spatial scale of the habitat. It is important to distinguish between the ecological scale (a scale at which a pattern or process occurs), observational scale (scale of the data being collected, for example, spatial resolution), and analytical scale (resolution of the method of analysis) (Lechner, Langford, Jones, Bekessy, & Gordon, 2012), and to explicitly report the details of each scale type. In heterogeneous environments, a random or even distribution of sampling effort results in high sampling variance, whereas stratifying the sampling by subhabitat or depth increases data precision (Underwood & Chapman, 2013). For previously mapped habitats, sampling efforts can also be concentrated within habitat boundaries (see Box ).

Within sampling strata, smaller, more numerous sampling units are better at estimating cover or detecting change than a few large units (Benedetti‐Cecchi, Airoldi, Abbiati, & Cinelli, 1996; Jokiel et al., 2015), similarly with scoring fewer points within many quadrats versus many points in a few quadrats (Drummond & Connell, 2005; Perkins, Foster, Hill, & Barrett, 2016), due to spatial variability and autocorrelation. Because sampling several replicates over a broad area is both time‐ and labor‐intensive, fixed transects have been used for assessing habitat status (Short et al., 2014). However, small differences in fixed transect placement (within centimeters to meters) can contribute to considerable variation in cover estimates (Davidson, 1997). Noninvasive markers along the transect routes can facilitate consistency in area captured, reducing this source of error. When establishing fixed transects, habitat heterogeneity must be accounted for to verify that trends observed along the transects will be similar to those occurring across the rest of the habitat.

#### 2.6.2 Resource‐driven design aspects

The depth and spatial scale of the biogenic habitat strongly influence resource‐driven design elements researchers must consider. For example, a major consideration of in situ fine‐scale manual surveys in shallow and intertidal habitats is the availability and expertise of adequately trained samplers, while broad‐scale surveys in deep‐water habitats are often limited by the high costs of vessel operation and gear deployment. It is critically important to ensure that despite resource limitations, ecological, observational, and analytical scales are matched appropriately.

### 2.7 Protocol review

In the process of finalizing method selection and sampling design, the survey protocol should be reviewed to check whether survey data will address monitoring objectives and whether additional resources could be saved. For example, surveys can be coordinated with existing monitoring efforts or utilize opportunistic data procured from fisheries bycatch (Durán Muñoz et al., 2011). For time and cost savings in sampling designs, high‐resolution data can be collected in temporal or spatial subsets, such as combining annual manual transects and decadal remote sensing surveys, or only recording species information from one in ten quadrats. Other considerations include whether data need to be accessible and comparable to other studies, for meta‐analyses or long‐term trends analyses. For long‐term monitoring, it is unlikely that same survey team will be retained for the duration of the program, and thus data collection and processing should be designed such that it is straightforward to train new surveyors and transfer relevant skills and knowledge. Additionally, given the propensity for short‐term conservation grants, long‐term monitoring requires judicious planning to ensure that survey methods are sufficiently cheap and logistically flexible to last through multiple iterations of project funding.

## 3 CONCLUSIONS

In oceans impacted by human pressures, biogenic habitat assessment and monitoring are crucial for attributing causes of decline and for providing solutions to mitigate habitat damage from anthropogenic impacts and monitoring environmental change (Downs, Woodley, Richmond, Lanning, & Owen, 2005). Systematic monitoring approaches, as laid out here, are urgently required to implement science‐based management, evaluate the success of protective measures, and guide adaptive management strategies for data‐limited marine biogenic habitats.

## CONFLICT OF INTEREST

None declared.

## AUTHORS' CONTRIBUTIONS

A. Dunham conceptualized paper, wrote case study, and made revisions; T.‐L. Loh conducted literature review and synthesis, wrote paper, and made revisions; and S.K. Archer wrote case study and made revisions.

## Data Availability

Analyses reported in this paper can be reproduced using the information provided in the paper and Appendix S1.
